# Targeting IFN activity to both B cells and plasmacytoid dendritic cells induces a robust tolerogenic response and protection against EAE

**DOI:** 10.1038/s41598-021-00891-6

**Published:** 2021-11-03

**Authors:** Anje Cauwels, Sandra Van Lint, Elke Rogge, Annick Verhee, Bram Van Den Eeckhout, Shengru Pang, Marco Prinz, Niko Kley, Gilles Uzé, Jan Tavernier

**Affiliations:** 1 Cytokine Receptor Laboratory, VIB Medical Biotechnology Center, Ghent University, A. Baertsoenkaai 3, 9000 Ghent, Belgium; 2 Institute of Neuropathology, Faculty of Medicine, University of Freiburg, 79106 Freiburg, Germany; 3 CNRS UMR 5235, University Montpellier, 34095 Montpellier, France; 4 BIOSS Centre for Biological Signalling Studies, University of Freiburg, 79106 Freiburg, Germany; 5 Orionis Biosciences, 9052 Ghent, Belgium

**Keywords:** Biotechnology, Immunology, Molecular medicine, Neurology

## Abstract

Type I Interferon (IFN) was the very first drug approved for the treatment of Multiple Sclerosis (MS), and is still frequently used as a first line therapy. However, systemic IFN also causes considerable side effects, affecting therapy adherence and dose escalation. In addition, the mechanism of action of IFN in MS is multifactorial and still not completely understood. Using AcTaferons (Activity-on-Target IFNs, AFNs), optimized IFN-based immunocytokines that allow cell-specific targeting, we have previously demonstrated that specific targeting of IFN activity to dendritic cells (DCs) can protect against experimental autoimmune encephalitis (EAE), inducing in vivo tolerogenic protective effects, evidenced by increased indoleamine-2,3-dioxygenase (IDO) and transforming growth factor β (TGFβ) release by plasmacytoid (p) DCs and improved immunosuppressive capacity of regulatory T and B cells. We here report that targeting type I IFN activity specifically towards B cells also provides strong protection against EAE, and that targeting pDCs using SiglecH-AFN can significantly add to this protective effect. The superior protection achieved by simultaneous targeting of both B lymphocytes and pDCs correlated with improved IL-10 responses in B cells and conventional cDCs, and with a previously unseen very robust IDO response in several cells, including all B and T lymphocytes, cDC1 and cDC2.

## Introduction

Multiple sclerosis (MS) is a chronic inflammatory autoimmune disease of the central nervous system (CNS). It is caused by an immune-mediated attack on the axonal myelin sheath, leading to debilitating brain and spinal cord damage. What exactly triggers MS is not completely understood, and is probably the combined result of genetic predisposition, environmental and viral factors^1^. The majority of MS patients are women, who experience their first symptoms between the ages of 20 and 40. For about 85% of these patients, MS starts with relapsing–remitting (RR) short episodes of worsening functions. If untreated, about half of these RR-MS patients will transition to secondary progressive (SP) MS within a decade of diagnosis. Worldwide, more than 2 million people are suffering from MS, and the prevalence seems to increase with latitude^1^. There are, however, several exceptions to this equatorial-polar gradient, such as very low rates in Canadian Inuit, Scandinavian Lapps and New Zealand Maori, and remarkably high rates in Sardinians and Palestinians^2,3^.

First line RR-MS treatment consisted for a long time only of type I IFN (IFN) and Glatiramer Acetate. More recently, oral drugs (Fingolimod, Teriflunomide, Dimethyl Fumarate) have gained significant importance as disease modifying therapies (DMTs)^1,4^. However, their exact modes of action are incompletely understood, and side effects including itching, alopecia, digestive problems and liver toxicity are frequent. During recent years, we have also seen the approval of several very successful antibody treatments, mainly used as second-line DMTs. These include Natalizumab (anti-VLA4), Alemtuzumab (anti-CD52), Daclizumab (anti-CD25) and Ocrelizumab (anti-CD20). These antibody therapies usually display better and longer-lasting effectiveness^1,4^. However, they also come with higher safety concerns, and Daclizumab was recently even withdrawn after reports of severe liver damage and inflammatory (meningo)encephalitis^5^.

In general, B cell depletion, although originally excluded from being promising based on negative preclinical mouse models, seems to be correlated with the best protection^6^. This may be achieved by anti-CD20 therapies such as Ocrelizumab, Rituximab or Ofatumumab, but also Alemtuzumab (anti-CD52) and the recently approved deoxyadenosine analog Cladribine/Mavenclad have been shown to efficiently deplete both B and T cells, followed by a rapid and selective repopulation with immature and mature B cells, while the disease-provoking memory B cells remain gone^7^. In retrospect, also the efficacy of other successful therapies, including that of IFN, seems to be correlated with memory B cell depletion^8,9^. Whether or not these new and promising B cell depleting strategies will be compromised by increased risks (inflammatory as well as oncologic) remains to be seen.

Another strategy that recently has gained interest is the treatment with autologous ex vivo generated tolerogenic dendritic cells (tolDC), not only for the treatment of MS, but also for other auto-immune diseases such as rheumatoid arthritis (RA), type I diabetes (T1D) and Crohn’s disease (CD)^10–12^. To generate these tolDCs, immunosuppressive agents are used to confer a tolerogenic phenotype to ex vivo generated DCs. These include vitamin D3, rapamycin, corticosteroids or immunosuppressive cytokines such interleukin-10 (IL-10) and TGFβ. Intriguingly, also pro-inflammatory cytokines may have the capacity to induce DC tolerance, such as IFN and TNF^13^.

IFN was the very first DMT to be approved for MS. Despite more than 25 years of use, its exact mechanisms of action and cellular targets remain largely unknown. In addition, IFN treatment causes multiple side effects, including flu-like symptoms, leukopenia, liver damage and depression^4,14,15^. Furthermore, up to 50% of MS patients are unresponsive to IFN, and in a subset of patients IFN treatment even induces relapses^16,17^. One possible explanation for this could be a mixed cellular response, where IFN signaling in certain cell types has a protective disease-delaying effect, while in other cell types it has a harmful disease-amplifying effect. Indeed, we have recently shown significant protection against EAE development by specific delivery of IFN activity to dendritic cells (DCs), whereas delivery to CD8^+^ cells was rather detrimental^18^. To cell-specifically deliver IFN activity, we use AcTaferons (AFNs), which consist of mutant type I IFN coupled to single domain antibodies (sdAbs) or ligands selectively recognizing cell-specific surface markers^19^.

In light of the recent successes reported with B cell depleting strategies in MS patients^6,7^, in combination with the inability of IFN to dampen EAE progression in mice in the absence of B cells^20^, we decided to compare targeting of IFN activity towards plasmacytoid DC (pDC) and/or conventional type 1 DC (cDC1) with B cell targeting. We found that B cell targeting can be superior to DC targeting, and that the specific combination of pDC and B cell targeting provides even better results than the individual treatments. This superior protection by type I IFN signaling in pDCs plus B lymphocytes is correlated with enhanced IL-10 expression in B cells and cDCs, as well as with IDO expression in all B and T lymphocytes and in type 1 and type 2 conventional DCs.

## Results

### Targeting IFN activity to B cells protects without toxicity

**Figure 1:**
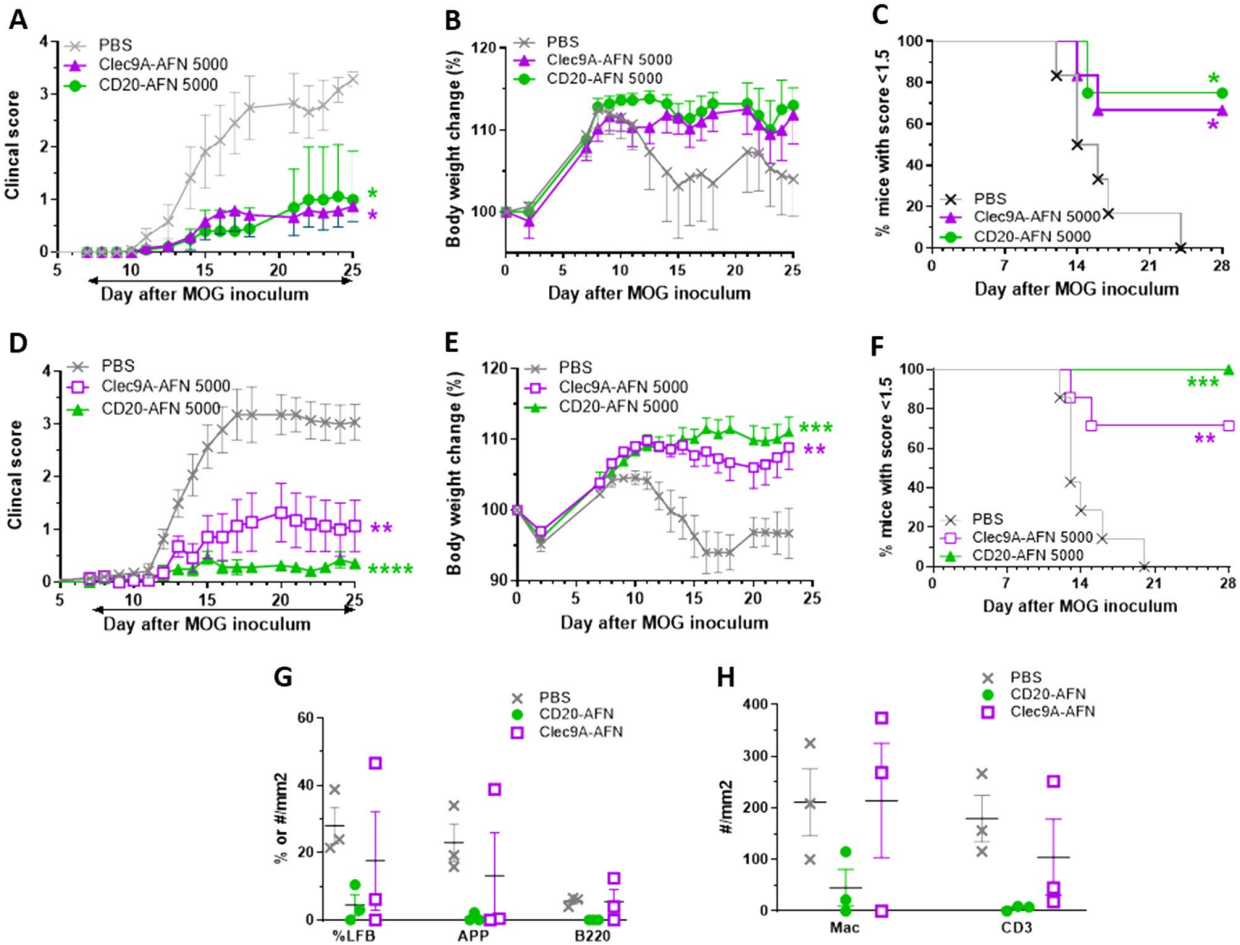
CD20-AFN protects at least as efficient as Clec9A-AFN against EAE progression. Shown are clinical scores (**A**,**D**), body weight (**B**,**E**), % of diseased mice (**C**,**F**) and spinal cord analysis (**G**,**H**) to evaluate demyelination (LFB), axonal damage (APP), B cells (B220), macrophages (Mac) and T cells (CD3). Shown are representative experiments [n = 6–7 for (**A**–**F**), n = 3 for (**G**,**H**)]. The black horizontal arrow indicates the daily treatment period, starting on d7, with 5000 IU AFNs. Differences were assessed using two-way ANOVA followed by Dunnett's multiple-comparison test (**A**,**B**,**D**,**E**), or using Chi Square Log-Rank test for the Kaplan–Meier plots (**C**,**F**); *P < 0.05, **P < 0.01, ***P < 0.001, ****P < 0.0001, compared with PBS treated animals.

The active EAE model using C57BL/6 mice immunized with MOG35-55 peptide is very robust and uniform and widely employed for understanding disease pathology and validating potential novel treatments^21^. In this model, we have previously shown good protection with DC-targeted AFNs, for which we used either Clec9A-AFN or SiglecH-AFN, the former being superior thanks to targeting of both pDCs and cDC1s^18^. SiglecH, in contrast to Clec9A, is only present on pDCs in mice^22^. In the current study, we treated EAE mice daily, starting on day 7 after MOG inoculation, with either PBS or 5000 IU of CD20-AFN, or 5000 IU Clec9A-AFN for comparison, and monitored clinical scores and body weight loss. Depending on the experiment, targeting B cells with CD20-AFN was consistently either as efficient (Fig. 1A–C), or sometimes even more efficient than DC-targeted AFN (Fig. 1D–F). Immunohistochemical analysis of spinal cords confirmed that CD20-AFN could better prevent neuronal damage and CNS inflammation compared to Clec9A-AFN (Fig. 1G–H).

Importantly, targeting B cells was not accompanied by the severe hematological deficits generally observed by high dose WT mIFN therapy, which we have previously shown to be mildly efficient to delay disease progression^18^ (Supplementary Figure). There is, however, a minor lymphopenia induced by CD20-AFN. This is due to a partial depletion of B lymphocytes by CD20-AFN therapy, as shown before^23^.

**Figure 2:**
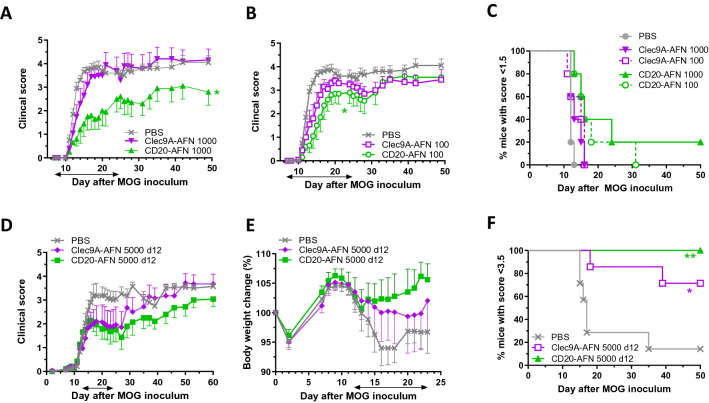
CD20-AFN protects better than Clec9A-AFN at lower doses, and in a therapeutic setting. Shown are clinical scores (**A**,**B**,**D**), % of diseased mice (**C**), body weight (**E**), or % of severely paralyzed mice (**F**). (**A**–**C**) Mice were treated with 1000 or 100 IU AFNs starting d7, (**D**–**F**) mice were treated with 5000 IU AFNs from d12 on. Shown are representative experiments (n = 5–7). The black horizontal arrow indicates the treatment period. Differences were assessed using two-way ANOVA followed by Dunnett's multiple-comparison test (**A**,**B**,**D**,**E**), or using Chi Square Log-Rank test for the Kaplan–Meier plots (**C**,**F**); *P < 0.05, **P < 0.01, compared with PBS treated animals.

Reducing the dose of AFN from 5000 to 1000 IU (Fig. 2A,C) or 100 IU (Fig. 2B,C) underscored the better effect of B cell targeting over DC targeting. In addition, 5000 IU of B cell targeted CD20-AFN also prevented disease progression when therapy was started after onset of disease, on day 12, just like DC targeting^18^, but also in this therapeutic setting, B cell targeting was better than DC targeting (Fig. 2D–F). Indeed, CD20-AFN therapy led to a rapid increase in body weight, whereas DC-targeting dampened the body weight loss rather than reverting it (Fig. 2E). In addition, upon cessation of AFN therapy, CD20-AFN treated mice reverted to progressive disease less rapidly (Fig. 2D) and were less prone to develop severe paralysis (Fig. 2F).

### Targeting IFN activity to both B cells and pDCs results in superior protection

**Figure 3:**
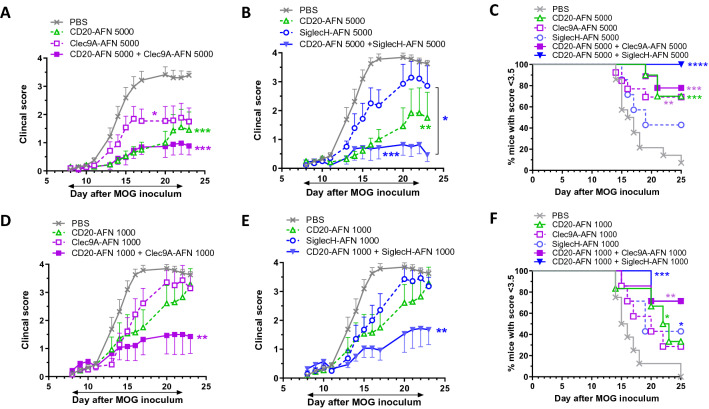
CD20-AFN protects best in combination with SiglecH-AFN therapy. Shown are clinical scores (**A**,**B**,**D**,**E**), or % of severely paralyzed mice (**C**,**F**). (**A**–**C**) Mice were treated with 5000 IU AFNs starting d8, (**D**–**F**) mice were treated with 1000 IU AFNs from d8 on. Shown are 2 pooled experiments (n = 10–12). The black horizontal arrow indicates the treatment period, starting on d8. Differences were assessed using two-way ANOVA followed by Tukey’s multiple-comparison test (**A**,**B**,**D**,**E**), or using Chi Square Log-Rank test for the Kaplan–Meier plots (**C**,**F**); *P < 0.05, **P < 0.01, ***P < 0.001, ****P < 0.0001, compared with PBS treated animals, unless otherwise indicated (**B**).

As we already published recently, the DC type that needs to be primarily stimulated to prevent disease progression are the pDCs, specifically targeted using SiglecH-AFN^18,22^. Later during disease progression, extra cDC1 targeting via Clec9A-AFN or XCL1-AFN adds to the protection^18^. In addition, we found that targeting DCs increased the tolerogenic capacity (IL-10 and TGFβ production) of regulatory T and B lymphocytes. To get an idea about the convergence or redundancy of DC- and B cell targeting effects, we next compared monotherapies with combined therapies. Similar to shown in Fig. 1D, targeting B cells with CD20-AFN had a better efficacy than targeting DCs with either Clec9A-AFN or SiglecH-ANF (Fig. 3A,B). In addition, at a dose of 5000 IU, it was clear that while Clec9A-AFN cannot add to the CD20-AFN-mediated protection, SiglecH-AFN boosted the protective capacity of CD20-AFN (Fig. 3A–C). Lowering the treatment dose from 5000 to 1000 IU, the only significant protection obtained was when these monotherapies were combined (Fig. 3D–F).

### Targeting B lymphocytes together with pDCs induces a superior systemic tolerogenic response

**Figure 4:**
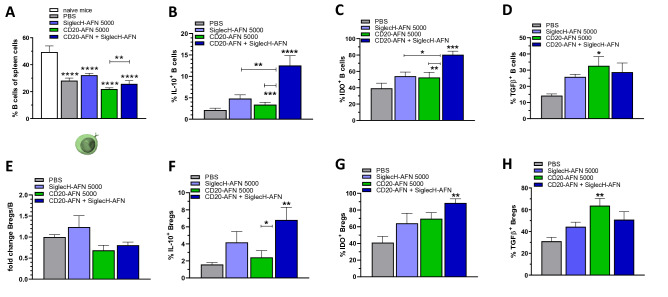
Superior protective combination therapy correlates with IL-10 and IDO expression in B cells. Amounts of splenic B cells were decreased on d15 due to EAE, AFN treatments did not affect that (**A**). IL-10 and IDO expression were dramatically increased if EAE mice were treated with the combination therapy (**B**,**C**). TGFβ was also increased, but only significantly by CD20-AFN monotherapy. Similar results were found in CD5^+^ CD1d^+^ Bregs, whose numbers were not affected by AFN therapies (**E**–**H**). Shown are 2 pooled experiments (n = 7). Differences were assessed using one-way ANOVA followed by Tukey’s multiple-comparison test; *P < 0.05, **P < 0.01, ***P < 0.001, ****P < 0.0001 compared with PBS treated animals, unless otherwise indicated.

To ascertain tolerance against self, cell types such as tolDCs, Tregs and Bregs are essential. To get an idea about the mechanism(s) involved in EAE protection by combined pDC and B cell targeting, we decided to analyze these cells for their tolerogenic potential. Unfortunately, we could not analyze pDCs, which were undetectable via SiglecH-dependent flow cytometry analysis after 8 daily treatments with SiglecH-AFN, due to the endocytic nature of SiglecH which is very efficiently internalized after engagement^24^. When we analyzed B lymphocytes 2 h after the 8th AFN or PBS treatment (on day 15 after MOG inoculation), we found that the CD19^+^ B cell population was decreased during EAE, and this was not significantly affected by mono- or combined therapies (Fig. 4A). Interestingly, the fully protective CD20-AFN + SiglecH-AFN combination therapy was the only therapy that significantly increased both IL-10 (six-fold) and IDO expression (two-fold) in the general B cell population (Fig. 4B,C). While TGFβ-positive B cells were increased by all therapies, this was only significant for CD20-AFN (Fig. 4D). Several B cell subtypes have been implied as potentially regulatory or tolerogenic, and CD5^+^ CD1d^+^ B lymphocytes are often referred to as Bregs. Exacerbated EAE progression due to B cell depletion before disease initiation can be corrected by the adoptive transfer of spleen CD1d^hi^ CD5^+^ Bregs, clearly demonstrating their tolerogenic efficacy^25^. Hence, we analyzed the numbers of CD5^+^ CD1d^+^ Bregs in spleens of EAE mice treated with AFNs, but could not find any significant differences in numbers (Fig. 4E). In terms of tolerogenic capacity, the Breg results were comparable to those obtained for the entire B cell population: significant increase was observed for IL-10 (4×) and IDO (2×) in mice that received the combination therapy (Fig. 4F,G), and for TGFβ in mice treated with CD20-AFN (Fig. 4H).

**Figure 5:**
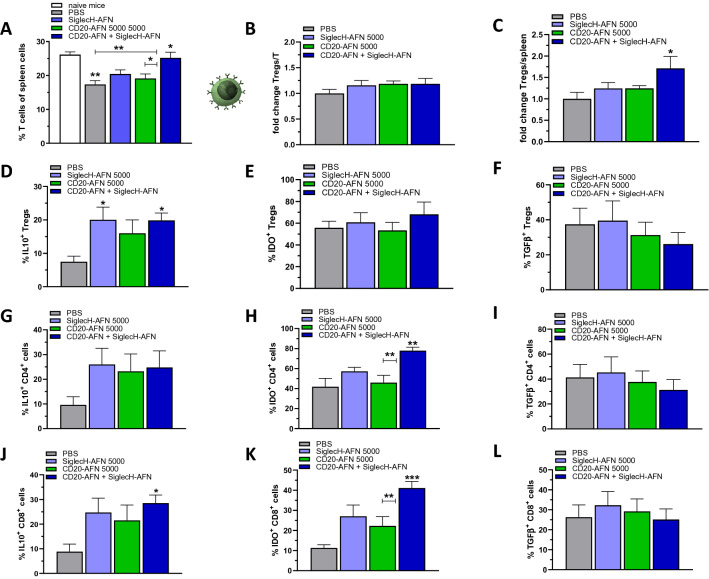
Superior protective combination therapy correlates with IL-10 and IDO expression in T cells. Amounts of splenic T cells were decreased on d15 due to EAE, monotherapy AFNs did not affect that but the combination therapy did (**A**). The relative amounts of Tregs on all T cells were not changed (**B**), but in the whole spleen cell population significantly more Tregs were present in case of combination therapy (**C**). CD4^+^ CD25^+^ FoxP3^+^ Tregs produced more IL-10 if treated with SiglecH-AFN (**D**), but IDO and TGFβ expression were not affected (**E**,**F**). In all CD4^+^ T cells (**G**–**I**) IL-10 expression tended to be increased by all therapies, IDO expression was significantly enhanced only if EAE mice were treated with the combination therapy, while TGFβ levels were not altered. In CD8^+^ T cells (**J**–**L**) IL-10 and IDO expression were increased in case of combination therapy, TGFβ levels were not altered. Shown are 2 pooled experiments (n = 7). Differences were assessed using one-way ANOVA followed by Tukey’s multiple-comparison test; *P < 0.05, **P < 0.01, ***P < 0.001, compared with PBS treated animals, unless otherwise indicated.

Next we analyzed the T cell populations. In general, CD3^+^ T lymphocytes were significantly decreased due to EAE (Fig. 5A). While monotherapies slightly improved T cell numbers, only the combined CD20-AFN + SiglecH-AFN therapy completely restored T cell numbers. Within the general CD3^+^ T cell population, CD4^+^ CD25^+^ FoxP3^+^ Tregs were not increased (Fig. 5B), but when analyzed in the entire spleen cell population they were enhanced in case of combination therapy only (Fig. 5C). Tregs produced more IL-10 if treated with SiglecH-AFN (Fig. 5D), but no differences in IDO or TGFβ could be observed (Fig. 5E,F). Surprisingly, however, when analyzing the entire CD4^+^ and CD8^+^ T cell populations, we found that they displayed elevated IL-10 levels (Fig. 5G,J), and especially their IDO expression was very significantly affected by the combination therapy (Fig. 5H,K). For CD4^+^ T cells, this resulted in a two-fold increase (Fig. 5H), for CD8^+^ T cells even in a four-fold increase (Fig. 5K). There was no increase in TGFβ (Fig. 5I,L).

**Figure 6:**
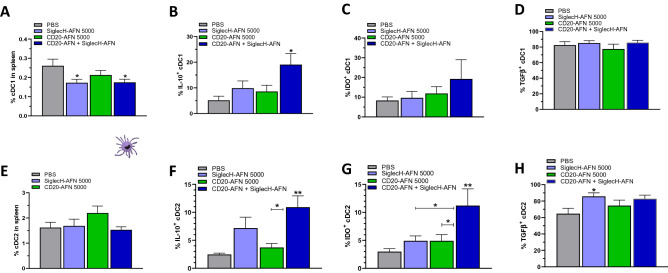
Superior protective combination therapy correlates with IL-10 and IDO expression in conventional dendritic cells. Numbers of splenic cDC1 decreased if EAE mice were treated with SiglecH-AFN (**A**), IL-10 expression was significantly enhanced only in case of combination therapy (**B**), and there were no significant changes in IDO or TGFβ expression (**C**,**D**). Numbers of cDC2 in EAE mice were not affected by AFN therapy with (**E**), IL-10 and IDO expression were significantly enhanced only in case of combination therapy (**F**,**G**), TGFβ expression by SiglecH-AFN only (**H**). Shown are 2 pooled experiments (n = 7). Differences were assessed using one-way ANOVA followed by Tukey’s multiple-comparison test; *P < 0.05, **P < 0.01, compared with PBS treated animals, unless otherwise indicated.

In our previous study, targeting DCs with SiglecH-AFN or Clec9A-AFN, we could not find any evidence at all of tolerization in conventional cDC1s on d12 (after 5 treatments) after MOG inoculation^18^. In our current study, we analyzed cDCs at a later time point and after 3 extra treatments (d15) and found evidence for a mild decrease in cDC1, but no changes in cDC2 (Fig. 6A,E). Interestingly, while monotherapies with either SiglecH-AFN or CD20-AFN could not significantly induce IL-10 or IDO in conventional DCs, the combined therapy resulted in dramatically enhanced levels (four- to five-fold increase) of IL-10 in both type 1 and type 2 cDCs (Fig. 6B,F)) and of IDO in cDC2s (Fig. 6G). IDO levels in cDC1s were not significantly increased after the combination therapy (Fig. 6C), and neither were TGFβ levels in cDC1s or cDC2s (Fig. 6D,H).

## Discussion

In a previous study, we have demonstrated that targeting type I IFN activity specifically to DCs can protect against EAE onset and progression. To achieve this, we used Clec9A-AFN (targeting both pDCs and cDC1s) and SiglecH-AFN (targeting pDCs only); long-lasting protection was best when Clec9A-AFN was used, or when SiglecH-AFN treatment was supplemented with XCL1-AFN (targeting cDC1s only) later during disease^18^. In the study reported here, we show that also targeting B cells with CD20-AFN is at least as protective as Clec9A-AFN, even when given after disease onset.

To evaluate the redundancy of DC and B cell targeting, we combined CD20-AFN with either Clec9A-AFN or SiglecH-AFN and found significant improvement of the therapy when B cell targeting was combined with pDC targeting. To understand the reason for this superior protection, we analyzed several cell types, including tolDCs, Tregs and Bregs, that have been implicated in inducing and maintaining tolerance against self. Mechanistically, cytokines such as IL-10 and TGFβ, as well as the induction of indoleamine 2,3-dioxygenase (IDO) activity, are known to be possibly involved in immunosuppression and tolerance. IL-10 is often referred to as the strongest immunosuppressive cytokine. Amongst others, it has a negative effect on APC and effector T cells, and a positive effect on regulatory T and B cells. In our previous study, we noted increased IL-10 production in Tregs and Bregs after 5 daily treatments with DC-targeted AFNs, but not in pDC or cDC1 populations^18^. TGFβ is another important immunosuppressive cytokine, which we found increased in pDCs, Tregs and Bregs early (on d12, after 5 treatments) during DC-targeted therapy^18^. IDO expression by APC is essential for fetal, oral, intestinal and transplant tolerance, as well as for dampening dangerous autoimmune activity in general^26^. Just like IL-10-deficient mice, IDO-deficient animals suffer from exacerbated EAE^27,28^. For IDO, immune cells have been identified to be responsible for this exacerbation, and the protective capacity of pDC transfer is largely dependent on their IDO expression^29^. IDO catalyzes the degradation of the essential amino acid tryptophan into catabolic products termed kynurenines. The reasons why IDO is often regarded as the major immunosuppressive mechanism are multiple^30^: (1) tryptophan depletion promotes anergy in CD8^+^ effector T cells and has an inhibitory effect on TH1 and TH17 differentiation and proliferation, (2) producing kynurenines that bind to the aryl hydrocarbon receptor (AhR), IDO activity results in Treg conversion and enhanced immunosuppressive Treg functions, plus in an additional feedforward IDO induction in DCs (“infectious tolerance”)^26^, and (3) a “moonlighting” signaling function next to its enzymatic function has been shown to upregulate its own expression as well as NF-κB signaling and TGFβ production^31,32^. Specifically in an EAE setting, pDCs have previously been shown to be the sole IDO expressing cells in lymph nodes, in contrast to IDO-negative cDCs, macrophages and B cells^29^, and we know from our previous study that SiglecH-AFN can induce significant IDO expression in pDCs^18^.

**Figure 7:**
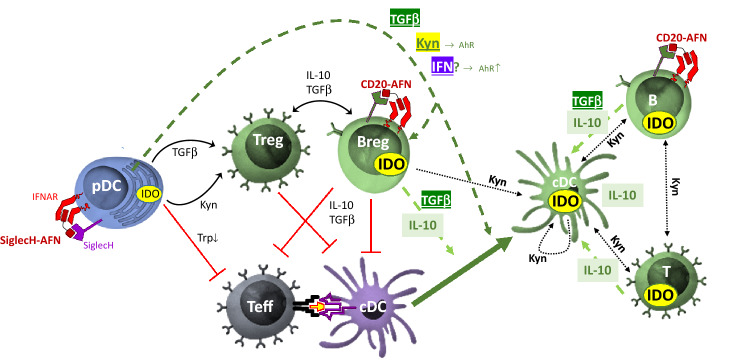
Graphical representation of possible tolerogenic cellular interactions induced by AFN targeting to pDCs and B lymphocytes. Targeting AFN via SiglecH to pDCs increases their TGFβ and IDO expression^18^. TGFβ and IDO-induced kynurenine synthesis can induce Tregs, while IDO-induced tryptophan catabolism inhibits Teff. Tregs and Bregs may reciprocally increase their immunosuppressive effects via IL-10 and TGFβ. Extra targeting of B lymphocytes (including Bregs) using CD20-AFN increases their IL-10 and TGFβ production (pale green dashed line). Together with TGFβ and/or kynurenines, and/or possibly type I IFN released by pDCs (dark green dashed line), these may be responsible for the substantial increase in IDO observed not only in B cells, but also in T cells and conventional cDCs. Importantly, IDO-produced kynurenine is well-known for triggering paracrine “infectious tolerance”, spreading from one cell type to another (black dotted lines)^26^, and an autocrine IDO-kyn-Ahr-IDO loop may induce long-term maintenance of the tolerogenic phenotype of cDCs (black dotted loop)^36^.

Interestingly, the completely protective CD20-AFN + SiglecH-AFN combination therapy was the only therapy that significantly increased both IL-10 and IDO expression in Bregs as well as in the general B cell population. T lymphocytes decreased during EAE, and only the combined CD20-AFN + SiglecH-AFN therapy completely restored T cell numbers and increased Tregs. Remarkably, the combined therapy also significantly enhanced IL-10 and IDO expression in the entire CD4^+^ and CD8^+^ T cell populations, as well as in both type 1 and type 2 cDCs. While pDCs, in contrast to cDCs, macrophages or B cells, are the only expressors of IDO, required for Treg generation to prevent EAE development^29^, several reports have clearly indicated that also cDCs can confer EAE resistance if they become IDO-positive^33,34^. How exactly IDO expression in conventional DCs specifically needs targeting of AFN towards pDCs ánd B lymphocytes may have several explanations. Transcriptional regulation of IDO in myeloid cells is complex. For efficient long-lasting IDO induction and immune tolerance, the majority of data point to a “two-signal” requirement, with a powerful “signal one” required before “signal two” can impart full tryptophan catabolism^26^. Potential triggers for IDO induction include Toll-like Receptor (TLR) ligands or IFNs as a “first signal”, aided by a “second signal” such as IL-10, TGFβ or kynurenines that may be supplied in an autocrine or paracrine manner^26,35,36^. In addition, in response to TGFβ signaling, IDO has also been shown to act as an intracellular signal transducer necessary for the induction of a stable and long-term immune tolerance^31,32^. Interestingly, type I IFN signaling in astrocytes can reduce EAE inflammation and disease scores via a mechanism involving tryptophan metabolites and AhR-dependent signaling^37^. As such, one can imagine that endogenous type I IFN, which is known to be massively produced by pDCs, may contribute to AhR-dependent signaling and immunosuppression in the CNS (Fig. 7). In addition, we have previously shown increased TGFβ and IDO, but not IL-10, in pDCs targeted with AFN^18^. Hence, pDC-derived TGFβ, kynurenine and/or possibly also endogenous IFN, in combination with one or more endogenous B cell-derived signals such as IL-10^38^ or TGFβ^31,32^, may be responsible for the dramatically increased IDO levels we observed in both targeted (B lymphocytes) as well as non-targeted cell types (T lymphocytes and cDCs) during SiglecH-AFN + CD20-AFN combination therapy (Fig. 7). Furthermore, this “two-signal” requirement for stable IDO induction, with an immunogenic trigger such as TLR ligands or IFN constituting “signal one” and an immunosuppressive trigger such as IL-10, TGFβ or kynurenine as “signal two”, may potentially provide an explanation for the long-term enigmatic paradox concerning the detrimental versus protective role of IFNγ in MS and EAE, as IFNγ may possibly have differential effects on different cell types (such as autoreactive T cells versus DCs) and/or in different disease stages^39,40^.

In conclusion, we here demonstrate that, next to DC targeting, also specific B cell targeting with type I IFN activity can significantly prevent and delay EAE progression. Best protective effects were obtained when B cell targeting was combined with pDC targeting, using CD20-AFN plus SiglecH-AFN. This superior clinical effect was accompanied by enhanced IL-10 release in B cells and conventional cDCs, and by the previously unseen induction of IDO in B lymphocytes, CD4^+^ and CD8^+^ T lymphocytes and cDC2s. Collectively, these data underscore the maximal tolerogenic potential of targeting type I IFN activity towards both pDCs and B lymphocytes, and indicate that the major molecular players are IDO and possibly IL-10.

## Methods

### EAE model and treatments

All animal experiments followed the Federation of European Laboratory Animal Science Association (FELASA) guidelines and were approved by the Ethical Committee of Ghent University. The EAE model was performed as previously described^18^. Male 8 weeks old C57Bl/6J mice were immunized subcutaneously (s.c.) with 200 µg MOG35-55 in CFA containing 1 mg heat-killed *Mycobacterium tuberculosis.* Two hours and two days later 50 ng Pertussis Toxin was injected i.p. First signs of disease typically start on day 10–12. IFN or AFN intraperitoneal (i.p.) treatments were initiated on day 7 or 12 and lasted till day 23–25, depending on the experiment. Mice were weighed and scored daily. A score ranging from 0 to 2 indicates progressive tail paralysis, with 1.5 for a partially limp tail (which is considered the onset of disease), and 2 for a completely limp tail. Score 2.5 is given if the animal no longer spreads its hind toes, score 3 for a waddled walk. Scores above 3 indicate increasing paralysis, with 3.5 for partial and 4 for complete hind limb paralysis. If fore limb paralysis is evident, score 5 is given and the animal is euthanized. Euthanasia was executed by CO_2_ inhalation by trained personnel using appropriate technique and equipment, death was confirmed by ascertaining respiratory and cardiac arrest. Treatment groups number 5–7 mice, experiments were repeated at least once. Differences were assessed using one-way or two-way ANOVA followed by Dunnett's or Tukey’s multiple-comparison test. Survival curves were compared using the log-rank test. GraphPad Prism software was used for statistical analysis. All values depicted are mean ± s.e.m.; *P < 0.05, **P < 0.01, ***P < 0.001 and ****P < 0.0001 compared with PBS treated animals, unless otherwise indicated.

### AcTaferons

We generated sdAbs selectively binding mouse CD20, Clec9A or SiglecH to use as targeting moieties. For the generation of AFNs, our lead IFNα mutant in mice is hIFNα2Q124R, a human IFNα2 mutant breaching the cross-species barrier and thus only very weakly active on murine cells (1/100 vs WT mIFNα). When fused to a targeting moiety that binds a cell-specific surface marker, AFNs regain full activity on the targeted cells by local avidity-driven receptor binding^19^. The generation and purification of AFNs was described before^23,41^.

### Haematological and flow cytometry analysis

One day after the last treatment, blood was collected from the tail vein in EDTA-coated microvette tubes (Sarstedt), and analyzed in a Hemavet 950FS whole blood counter (Drew Scientific, Waterbury, USA). Spinal cord sections were dissected and stained with H&E, Luxol fast blue (LFB, for myelination assessment), and antibodies against amyloid precursor protein APP (evaluating axonal damage) or CD3, B220 or MAC3 for visualizing infiltrating T and B cells and macrophages, respectively^18^. Flow cytometry was done on spleen cells 15 days after MOG inoculation (two hours after the 8th treatment). Doublets were excluded and living cells were selected based on live-dead stain (Invitrogen). pDC (CD3^−^ CD19^−^ B220^+^ SiglecH^+^), cDC1 (CD3^−^ CD19^−^ CD11b^−^ CD11c^+^ MHCII^+^ XCR1^+^) and cDC2 (CD3^−^ CD19^−^ CD11b^+^ CD11c^+^ MHCII^+^ XCR1^−^) percentages were determined, and the intracellular expression of designated cytokines determined. For Tregs, the CD3^+^ CD4^+^ CD8^−^ CD25^+^ FoxP3^+^ population was analyzed. For Bregs, the CD19^+^ CD5^+^ CD1d^+^ population. Fc receptors were blocked using anti-CD16/CD32 Ab. Fluorescence minus one (FMO) controls were included to allow adequate analysis. Samples were acquired on an Attune Nxt Acoustic Focusing Cytometer (Life Technologies) and analyzed using FlowJo software.

### Ethical approval

All animal experiments followed the Federation of European Laboratory Animal Science Association guidelines and were approved by the Ethical Committee of the Faculty of Medicine, Ghent University, and carried out in compliance with the ARRIVE guidelines.

## Supplementary Information

Supplementary Information.
