# Supplementary material for: Targeting IFN activity to both B cells and plasmacytoid dendritic cells induces a robust tolerogenic response and protection against EAE
Source: Sci Rep. 2021 Nov 3;11:21575. doi: 10.1038/s41598-021-00891-6 (PMC8566508; doi:10.1038/s41598-021-00891-6)

Supplemental Figure

Daily CD20-AFN or Clec9A-AFN therapy (d7-25) does not cause hematological abnormalities. Shown are lymphocytes (A), neutrophils (B), red blood cells (C) and platelets (D) in circulation 1 day after the last treatment with 5000 IU AFN. For comparison, hematological parameters in mice treated with 5000 or 1.000.000 IU wild-type (WT) mIFN are added. Shown is a representative experiment (n = 6, except for the high dose WT-mIFN where only 2 mice survived the treatment). Differences were assessed using one-way ANOVA followed by Dunnett's multiple-comparison test; *P < 0.05, ***P < 0.01, ***P < 0.001 compared with PBS treated animals.

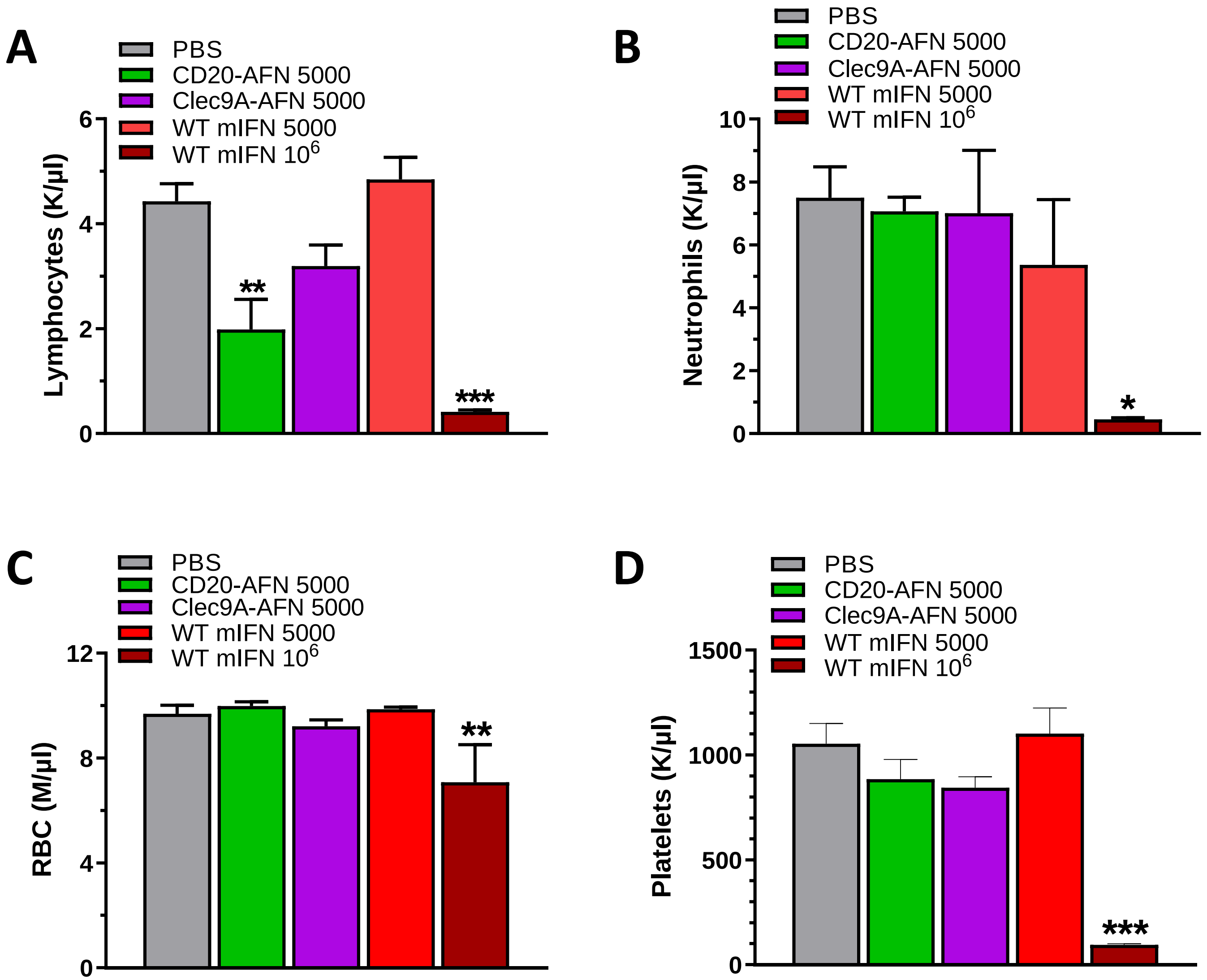

Supplement: Supplementary file 1 — Supplementary Information. [file 41598_2021_891_MOESM1_ESM.pdf]
